# Reproductive interference and sensitivity to female pheromones in males and females of two herbivorous mite species

**DOI:** 10.1007/s10493-020-00492-4

**Published:** 2020-04-19

**Authors:** Yukie Sato, Juan M. Alba

**Affiliations:** 1 Mountain Science Center, Faculty of Life and Environmental Sciences, University of Tsukuba, 1-1-1 Tennodai, Tsukuba, Ibaraki 305-8577 Japan; 2 Evolutionary and Population Biology-IBED, University of Amsterdam, P.O. Box 94240, 1090GE Amsterdam, The Netherlands

**Keywords:** Male mating preference, Reproductive interference, Web sharing, Pheromone, *Tetranychus evansi*, *Tetranychus urticae*, Tomato

## Abstract

Competitive interaction between sister species can be affected by reproductive interference (RI) depending on the ability of males to discriminate conspecific from heterospecific mates. We study such interactions in *Tetranychus evansi* and *T. urticae*. These spider mites co-occur on solanaceous plants in Southern Europe, and cause important yield losses in tomato crops. Previous studies using Spanish populations found that *T. evansi* outcompetes *T. urticae,* and that this is due to unidirectional RI of *T. evansi* males with *T. urticae* females. The unidirectional RI is attributed to differences in male mate preference for conspecific females between the two species. Also, differences in the propensity of interspecific web sharing in females plays a role. To investigate proximate mechanisms of this RI, here we study the role of female pheromones on male mate preference and female web sharing. We extracted pheromones from females of the two species, and investigated if males and females were arrested by the pheromone extractions in various concentrations. We observed that *T. urticae* males were more sensitive to the pheromone extractions and able to discriminate conspecific from heterospecific ones. *Tetranychus evansi* males, on the other hand, were less sensitive. Females from both species were arrested by conspecific pheromone extraction in lower concentrations. In conclusion, heterospecific mating by *T. evansi* males, which results in RI, can be explained by their lack of discrimination between female pheromones of the two species. Differences in the propensity of interspecific web sharing in females might not be explained by the pheromones that we investigated.

## Introduction

Closely related species typically compete for space and resources whenever they co-occur. Therefore, resource competition is often considered as the mechanism leading to spatial and temporal segregation among closely related species. Although such segregation is also found in arthropod herbivores, competitive exclusion for resources may play a minor role in arthropod herbivores due to the high abundance of plants (Kuno 1992). Instead, an important mechanism explaining their spatial and temporal segregation may be reproductive interference (RI), a negative sexual interaction between species (Kuno 1992; Burdfield-Steel and Shuker 2011; Ruokolainen and Hanski 2016, but see Shuker and Burdfield‐Steel 2017). For example, two congeneric species of groundhoppers, *Tetrix subulata* and *T. ceperoi*, broadly overlap in geographic distribution, yet rarely co-occur at the same site (Hochkirch et al. 2007). Males of *T. ceperoi* are attracted to *T. subulata* females due to their larger size compared to conspecifics females (Hochkirch et al. 2006). Although *T. subulata* females reject heterospecific mating, their reproductive success decreases when both species co-occur in the same habitat (Hochkirch et al. 2007). Therefore, RI is considered to be an important factor contributing to their spatial and temporal segregation. RI can be caused by various processes and mechanisms; however, the most common cause concerns misdirected mating attempts as in the case of *T. subulata* and *T. ceperoi* (Gröning and Hochkirch 2008; Burdfield-Steel and Shuker 2011; Shuker and Burdfield‐Steel 2017). Indeed, a male is often thought to be the less choosy sex. It is because males may increase fitness by mating with as many females as possible and suffer little reduction in fitness, even if mating with reproductively isolated heterospecific females (Bateman 1948; Noor 1996; Kozak et al. 2009; Willis 2013). However, it is known that males also use multiple cues to detect conspecific females and even show mate preference for specific females among conspecifics (Bonduriansky 2001; Edward and Chapman 2011). Therefore, to determine the cause of RI, it is important to know what kind of cues males use for searching and accepting females and how precisely males can detect appropriate females.

Recently, we found that RI affects competitive interaction between two congeneric spider mites. One is the tomato red spider mite, *Tetranychus evansi*, and the other is the two-spotted spider mite, *T. urticae*, both of which were collected from Spain (Sato et al. 2014a)*.* These spider mites are important pests of tomato plants, *Solanum lycopersicum* L., in Europe. They spin silken webs on the leaf surface of tomato plants, and feed, develop and reproduce under the protection of the web. *Tetranychus evansi* originates from South America; however, it has spread into Africa, Southern Europe and East Asia (Boubou et al. 2012; Navajas et al. 2012). As a result, both species co-occur on solanaceous plants in greenhouses and fields in Europe (Ferrero et al. 2011; Ferragut et al. 2013). Previous studies revealed that *T. urticae* takes advantage of *T. evansi* pre-infestation due to plant defence suppression (Sarmento et al. 2011a). However, when together on tomato, *T. evansi* outcompetes *T. urticae* (Sarmento et al. 2011b). This outcome is consistent with field observations in Spain, in which *T. evansi* became the most abundant species in non-crop fields where native *Tetranychus* species including *T. urticae* have been present (Ferragut et al. 2013). RI has been described in several species of spider mites, for example, between *Panonychus mori* and *P. citri* (Fujimoto et al. 1996; Takafuji et al. 1997) and between *T. urticae* and *T. turkestani* (Ben-David et al. 2009), and it is considered to be one of the mechanisms determining the outcome of interspecific competition. Besides, mating with incompatible mates has been often observed within and between species (e.g., Takafuji and Fujimoto 1985; Gotoh 1986; Sato et al. 2000a, b; Knegt et al. 2017) and even between genera (Collins and Margolies 1991). Therefore, we focused on the role of RI in their competitive interaction, and found that *T. urticae* failed to increase its population under the condition that RI works strongly (Sato et al. 2014a). The results were supported by crossing experiments using these populations. *Tetranychus evansi* males readily mate with *T. urticae* females, and even show strong mate preference for *T. urticae* females, while they are reproductively isolated: females of both species mated with heterospecific males produce only haploid males arising from unfertilized eggs (Sato et al. 2014a). On the other hand, *T. urticae* males do mate with *T. evansi* females; however, they show strong mate preference for conspecific females in choice conditions (Sato et al. 2014a). The difference in mate preference between *T. evansi* and *T. urticae* males may be a key factor in their competitive interaction because it causes unidirectional RI. The observed mate preferences in *T. evansi* and *T. urticae* males were replicated in the same populations and also in Brazilian populations (Sato et al. 2016b), but male mate preference for heterospecifics was not found in Portuguese populations (Clemente et al. 2016).

Male mate preference for heterospecifics in *T. evansi* may be surprising, because they cannot reproduce by mating with *T. urticae* (Sato et al. 2014a), and it can decrease mating opportunities with conspecifics. Costs of mating possibly exists, although it is unclear how many times males can mate in a day and in their life time, and how costly a mating is (Rodrigues et al. 2020). However, male mate preference for heterospecifics was also found in groundhoppers and geckos, probably caused by a side-effect of preference for larger females associated with higher fecundity (Dame and Petren 2006; Hochkirch et al. 2007). Similarly, preference for heterospecifics in *T. evansi* males may be a side-effect of preference for non-kin individuals, given that *T. urticae* males show preference for non-familiar individuals to avoid inbreeding (Yoshioka and Yano 2014). This idea was tested by comparing mate preference of *T. evansi* males for *T. urticae* females when the alternative conspecific female was kin or non-kin using Spanish and Brazilian populations. As predicted, in both Spanish and Brazilian populations, the propensity to mate with heterospecifics tended to be lower when the alternative conspecific females were non-kin, although significant differences were not detected (Sato et al. 2016b).

Female mate preference can also influence the result of RI between closely related species (Gröning and Hochkirch 2008). However, in spider mites, female mate preference has a minor role due to male guarding behaviour of teleiochrysalis females (females in their last moulting stage before emerging as adults). Guarding males help female moulting and typically copulate with the guarded female just after emergence (Potter et al. 1976). Even after emergence, females rarely succeeded in rejecting mating with heterospecific males (e.g., Sato et al. 2014a). Alternatively, in females, the gregariousness and propensity to share their web with different species may affect the likelihood of RI because these affect the probability that females meet heterospecific males. Heterospecific web sharing easily occurs because of the function of webs as shelters against predators (Yano 2012). We previously found that *T. evansi* females tend to avoid sharing webs with *T. urticae* females, whereas *T. urticae* females show a preference for sharing webs with *T. evansi* females (Sato et al. 2016a). In addition, *T. evansi* females show higher aggregation with conspecifics on a tomato plant than *T. urticae* females (Sato et al. 2016a). The documented difference in propensity of interspecific web sharing and gregariousness suggests that *T. evansi* females are less prone to RI compared to *T. urticae* females.

Here, to elucidate potential proximate mechanisms of RI, we focus on the role of female pheromones on mate preference in males as well as on interspecific web sharing in females in *T. evansi* and *T. urticae*. *Tetranychus urticae* males use sex pheromones to find females (Cone et al. 1971; Royalty et al. 1992, 1993a, b; Rasmy and Hussein 1994; Margolies and Collins 1994; Oku et al. 2015; Rodrigues et al. 2017). This behaviour has not been investigated in *T. evansi* but it has been reported in another genus of spider mites, *Oligonychus pratensis* (Margolies and Collins 1994). Differences in attractiveness of female pheromones and sensitivity to the chemicals between *T. evansi* and *T. urticae* can be associated with their difference in male mate preference. Besides, if males use female pheromone, females possibly use the pheromone for their decision of web sharing and also gregariousness. Therefore, in this study, we extract pheromones from *T. evansi* and *T. urticae* females, and investigate responses to the pheromone extractions in males and females of these two species.

## Materials and methods

### Mites

We used the populations *T. evansi* Algarrobo-1 and *T. urticae* Algarrobo-1 collected from *Solanum nigrum* L. in August 2010, in Málaga, Spain (N36°34′29″, W5°57′35″). These are the same populations used in Sato et al. (2014a) which exhibited the effect of RI on the competitive interaction between *T. evansi* and *T. urticae*. *Tetranychus urticae* was initially reared on detached common bean leaves (*Phaseolus vulgaris* L.), and subsequently reared on detached tomato leaves (cv. Castlemart) for more than two generations. *Tetranychus evansi* was reared on detached tomato leaves (cv. Castlemart). Detached leaves were placed on wet cotton wool in a plastic box in a climate room under constant conditions (25 °C; 60% RH; 16:8 h light: dark photoperiod) at the University of Amsterdam, the Netherlands. We collected teleiochrysalis males of *T. evansi* and *T. urticae* developed from eggs laid by isolated virgin females. Teleiochrysalis females were collected from the mite cultures. Collected teleiochrysalis males were isolated on tomato leaf discs (15 mm diameter, cv. Castlemart). Moulting stage was checked every 24 h. In the bioassay, we used males and females which emerged within 48 h and did not have any experience with other individuals.

### Pheromone extraction

In our method for pheromone extraction, we followed the method in previous studies (Royalty et al. 1992, 1993b; Margolies and Collins 1994), with a few small changes, based on a pilot experiment we performed. Previous studies reported that pheromones arresting adult males can be extracted from females in the protonymph, deutochrysalis, deutonymph, teleiochrysalis, and adult stage, as well as from adult males, and that extracts from teleiochrysalis females induce the highest male response and those from adult males induce the lowest male response (Royalty et al. 1992, 1993b; Margolies and Collins 1994). Therefore, we used teleiochrysalis females for the extraction of pheromone, even though we used adult females to assess male mate preference in our previous studies (Sato et al. 2014a, 2016b). Two hundred teleiochrysalis females were collected from the cultures of each species, and soaked into 1 mL hexane (Sigma-Aldrich, Germany) in a 2-mL glass tube for either 24 or 48 h, under constant climatic conditions (25 °C, 60% RH, 16:8 h light: dark photoperiod). In previous studies, mites were soaked for 24 to 120 h (Royalty et al. 1992, 1993b; Margolies and Collins 1994). Although such long extraction possibly results in extracting not only from the mite body surface but also from the inside, we chose 24 and 48 h extraction by following previous studies and also based on our pilot experiment. Tubes were mixed by inversion several times, then after mites had precipitated, 800 µL of the supernatant was transferred to a new vial. Extracts were stored at −20 °C until the performance of bioassays.

### Bioassay

Extracts of hexane in which females were soaked for 24 h (hereafter, 24 h-extract) were used in six treatments differing in extracted mite species (extracts from *T. urticae* females and from *T. evansi* females, and hexane as the control) and the concentration of extract (low and high) (Table 1). Extracts of hexane in which females were soaked for 48 h (hereafter, 48 h-extract) were used in three treatments differing in the extracted mite species (Table 1). Adult males and females of each species were used in the experiments.

**Table 1:** Treatments in bioassay using males and females of *Tetranychus evansi* and *T. urticae*

Type of extract	Extract duration	24 h						48 h		
	Concentration	Low (1 μL)			High (1 μL × 2)			Low (1 μL)		
Extracted mite		*T. evansi*	*T. urticae*	Control (hexane)	*T. evansi*	*T. urticae*	Control (hexane)	*T. evansi*	*T. urticae*	Control (hexane)
Set of trials		A	A	A	B	B	B	C	C	C

By using extracts in which females were soaked for 24 h, we carried out six treatments differing in extracted mite (*T. evansi*, *T. urticae* and hexane as control), and concentration (low and high). By using extracts in which females were soaked for 48 h, we carried out three treatments differing in extracted mite (*T. evansi*, *T. urticae* and hexane as control). As we carried out these treatments in *T. evansi* males, *T. urticae* males. *T. evansi* females and *T. urticae* females, respectively, there were 36 treatments in total. We carried out three treatments, in which type of extract (extract duration and concentration) and tested mite (*T. evansi* males, *T. urticae* males. *T. evansi* females and *T. urticae* females) were the same but the extracted mite (*T. evansi* females and *T. urticae* females) was different, at the same time as a set. Each panel in Figs. 3, 4, 5 and 6 shows the results in each set, and the capital letters in this table correspond to the figure panels

**Fig. 1:**
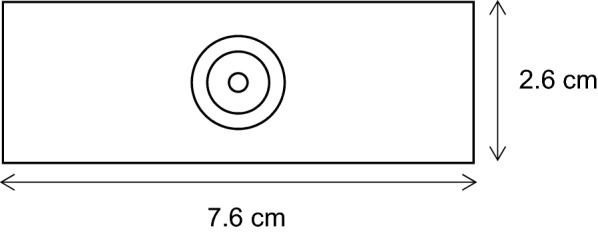
The arena used for the bioassay. A sheet of paper, on which there are three circles (3, 10 and 15 mm in diameter) was placed under a slide glass cleaned with ethanol. The smallest circle was used as the area for dropping extract, and the largest circle was used as the arena for observation of arrestment

Arrestment bioassays were performed on a slide glass (7.6 × 2.6 cm, Thermo-Scientific, USA) cleaned with ethanol. A marked paper with three circles (3, 10 and 15 mm diameter) was attached under the slide glass (Fig. 1). We dropped 1 µL of extract or hexane in the smallest circle (3 mm diameter) as the treatment of low concentration (equivalent to extract from 0.2 teleiochrysalis female). For treatment of high concentration, we dropped 1 µL of extract or hexane twice in the smallest circle not to make the liquids flow out of the circle (equivalent to 0.4 teleiochrysalis female). When we used much higher concentration in our pilot experiment, we observed that the extracts were sticky and mites were trapped by them. Therefore, we used these two concentrations in the experiment. Hexane evaporated at room temperature after which the arena was used for the bioassay. The arrestment bioassay was performed by placing an individual mite in the treated circle and recording the duration that the mite spent in the largest circle (15 mm diameter). In previous studies, 3.0 to 3.5 mm circles were used not only for extract depositing but also for arrestment observation, and observation was terminated when a mite left the circle more than 5 s (Royalty et al. 1992, 1993b; Margolies and Collins 1994). We tried the same procedure in our pilot experiments, but we found that mites kept walking and tapping their first legs as if they were searching the range and concentration of extracts, and frequently moved in and out of the circle. We also observed that when they moved away from the circle with the extract, they passed the half of the largest circle immediately (1–2 s). Therefore, we decided to use the largest circle (15 mm diameter) for the arrestment observation area and terminated our observation when they left the largest circle. We did not use the middle-sized circle (10 mm diameter), although there was a line on the bioassay arena. As time of the day may affect their activity, the trial start time was recorded also. We carried out three treatments at the same time as a set, in which the extracted mite species (*T. evansi*, *T. urticae* or hexane control) was different but the concentration, extraction time, and tested mite (species and sex) were the same (Table 1). For example, we measured the durations that *T. urticae* males were arrested by 1 µL of 24 h-extract from *T. urticae* females, 1 µL of 24 h-extract from *T. evansi* females, and 1 µL of hexane as a control in the same series. We carried out several sets, which are different in extract, concentration and tested mites, during 1 day in a mixed order. We carried out the experiment from 09:30 to 20:00 h for 14 days.

### Statistical analysis

The statistical analyses were performed with the statistical package R v.3.6.1 (R Core Team 2019). We analysed the duration of arrestment in males and females separately with tested mite (*T. evansi* or *T. urticae*), extracted mite (conspecific females, heterospecific females or the control), type of extract (low concentration of 24 h-extract, high concentration of 24 h-extract or low concentration of 48 h-extract), interactions of these three factors (tested mite × extracted mite × type of extract), trial start time and the interaction with tested mite (trial start time × tested mite) by using a generalized linear model (GLM) with gamma error distribution. Then, we performed stepwise model selection by Akaike's Information Criterion (AIC) to choose a model (*step* in the package *stats*; R Core Team 2019). To compare the duration of arrestment between treatments within each set, we performed multiple comparisons with Tukey method (*glht* in the package *multcomp*) (Hothorn et al. 2008) after constructing a GLM of duration of arrestment with gamma error distribution in each set.

## Results

**Fig. 2:**
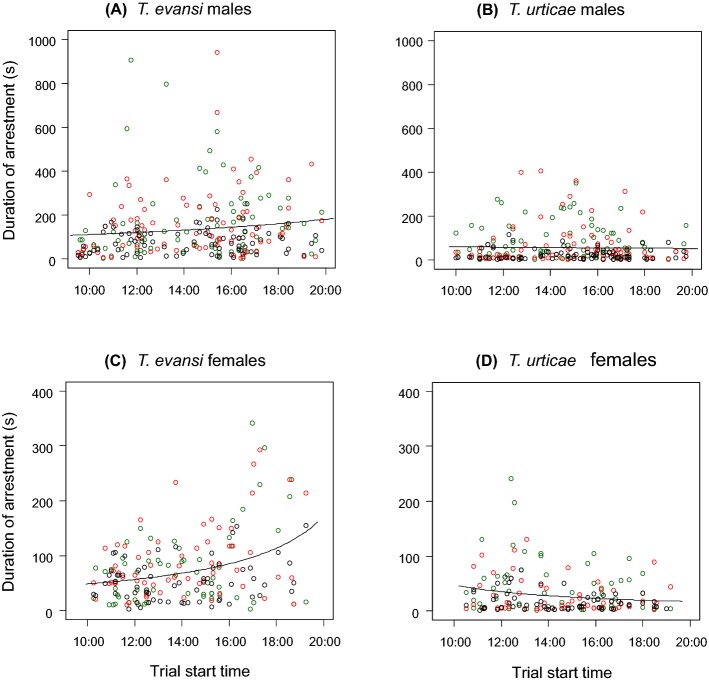
Relationship between trial start time and duration of arrestment in *Tetranychus evansi* males (**a**), *T. urticae* males (**b**), *T. evansi* females (**c**) and *T. urticae* females (**d**). Solid lines indicate the prediction from a generalized linear model (gamma error distribution) of duration of arrestment with trial start time in each mite type. Red, green and black circles indicate the treatments using extracts from *T. evansi* females, *T. urticae* females and the control (hexane), respectively. (Color figure online)

**Fig. 3:**
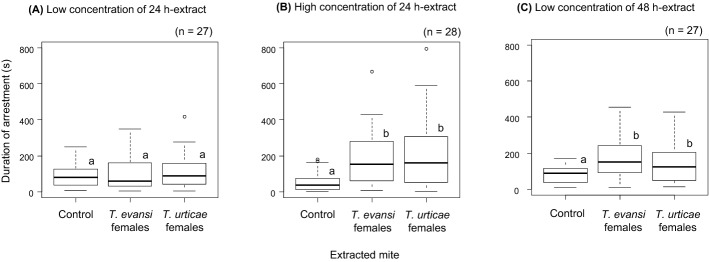
Box plots of the duration that *Tetranychus evansi* males were arrested by low concentration of 24 h-extract (**a**), high concentration of 24 h-extract (**b**) and low concentration of 48 h-extract (**c**)*.* The numbers in parentheses indicate the numbers of replicates. Different letters on the boxes show significant difference detected by multiple comparison with Tukey method (p < 0.05) after constructing a generalized linear model (gamma error distribution) with treatment and trial start time in each set

**Fig. 4:**
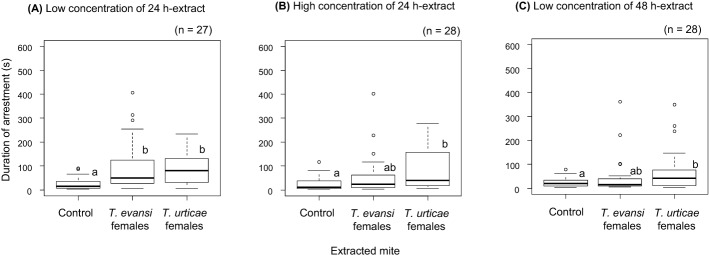
Box plots of the duration that *Tetranychus urticae* males were arrested by low concentration of 24 h-extract (**a**), high concentration of 24 h-extract (**b**) and low concentration of 48 h-extract (**c**). The numbers in parentheses indicate the numbers of replicates. Different letters on the boxes show the significant difference detected by multiple comparison with Tukey method (p < 0.05) after constructing a generalized linear model (gamma error distribution) with treatment and trial start time in each set

In males, the effects of interactions between tested mite and extracted mite and between tested mite and type of extract were significant, indicating that the effects of extracted mite and type of extract were different depending on the tested mite (Table 2a). Trial start time was not in the selected model, indicating that it did not have a significant effect on the duration of arrestment in males (Figs. 2a, b). Males of *T. evansi* were not arrested by the treatments with low concentration of 24 h-extract (Fig. 3a), but they were arrested by the extracts from both conspecific and heterospecific females in the treatments with high concentration of 24 h-extract and with 48 h-extract (Fig. 3b, c). Males of *T. urticae* were arrested by the extracts from both conspecific and heterospecific females in the treatment with low concentration of 24 h-extract (Fig. 4a), and they were arrested only by the extracts from conspecific females in the treatments with high concentration of 24 h-extract and with 48 h-extract (Fig. 4b, c).

**Table 2:** Analysis of deviance table of stepwise-selected model (a generalized linear model with gamma error distribution) of duration of arrestment in males (a) and females (b) in *Tetranychus evansi* and *T. urticae*

Explanatory variables	Df	Deviance	Residual Df	Residual deviance	F	p
(a) Males						
NULL			493	684.776		
Tested mite (TM)	1	90.486	492	594.290	81.744	<0.001
Extracted mite (EM)	2	59.686	490	534.604	26.960	< 0.001
Type of extract (TE)	2	2.209	488	532.395	0.998	0.37
TM × EM	2	23.074	486	509.321	10.423	< 0.001
TM × TE	2	7.659	484	501.661	3.460	0.032
(b) Females						
NULL			374	459.689		
Tested mite (TM)	1	82.125	373	377.564	97.971	< 0.001
Extracted mite (EM)	2	17.788	371	359.776	10.610	< 0.001
Type of extract (TE)	2	6.741	369	353.035	4.021	0.019
Trial start time (TST)	1	5.679	368	347.357	6.774	0.010
TM × EM	2	10.548	366	336.809	6.292	0.002
TM × TE	2	1.311	364	335.498	0.782	0.46
TM × TST	1	22.055	363	313.443	26.310	< 0.001

In the saturated models, the dependent variable is duration of arrestment, and the explanatory variables are tested mite (*T. evansi* or *T. urticae*), extracted mite (conspecific females, heterospecific females or the control), type of extract (low concentration of 24 h-extract, high concentration of 24 h-extract or low concentration of 48 h-extract), the interactions of these three factors, trial start time and the interaction between trial start time and tested mite

**Fig. 5:**
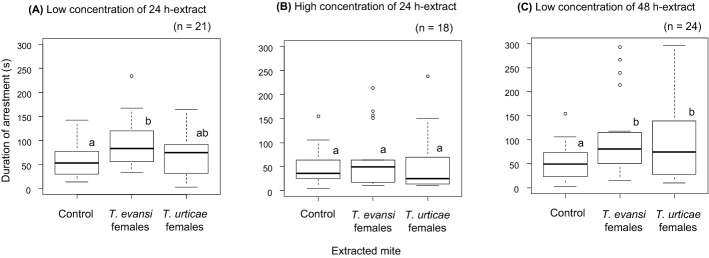
Box plots of the duration that *Tetranychus evansi* females were arrested by low concentration of 24 h-extract (**a**), high concentration of 24 h-extract (**b**) and low concentration of 48 h-extract (**c**)*.* The numbers in parentheses indicate the numbers of replicates. Different letters on the boxes show the significant difference detected by multiple comparison with Tukey method (p < 0.05) after constructing a generalized linear model (gamma error distribution) with treatment and trial start time in each set

**Fig. 6:**
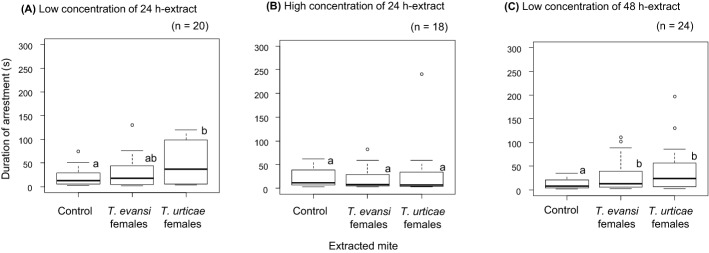
Box plots of the duration that *Tetranychus urticae* females were arrested by low concentration of 24 h-extract (**a**), high concentration of 24 h-extract (**b**) and low concentration of 48 h-extract (**c**)*.* The numbers in parentheses indicate the numbers of replicates. Different letters on the boxes show the significant difference detected by multiple comparison with Tukey method (p < 0.05) after constructing a generalized linear model (gamma error distribution) with treatment and trial start time in each set

In females, effects of interactions between tested mite and extracted mite, between tested mite and type of extract and between tested mite and trial start time were significant (Table 2b), indicating that the effects of extracted mite, type of extract and trial start time were different depending on the tested mite. The later the trials started in the day, the longer the arrestment duration in *T. evansi* females and the shorter in *T. urticae* females (Fig. 2c, d). Females of *T. evansi* and *T. urticae* were arrested by the extracts only from conspecific females in the treatment with low concentration of 24 h-extract (Figs. 5a, 6a); however, the effects disappeared in the treatment with high concentration of 24 h-extract (Figs. 5b, 6b). Females of *T. evansi* and *T. urticae* were arrested by the extracts both from conspecific and heterospecific females in the treatment with 48 h-extract (Figs. 5c, 6c).

## Discussion

### Sex pheromones and male mate preference

In previous studies, *T. urticae* males showed strong mate preference for conspecific females in choice conditions (Sato et al. 2014a, 2016b). In this study, we investigated if *T. urticae* males were arrested by pheromone extracts from *T. urticae* and *T. evansi* females by no-choice tests, and found that *T. urticae* males were arrested by female pheromones of either species when the concentration of extract was low (equivalent to 0.2 teleiochrysalis female in 24 h-extract). This result is consistent with the findings in the previous study focusing on reproductive relationships between *T. urticae* and *O. pratensis*: *T. urticae* and *O. pratensis* males were arrested by extracts both from conspecific and heterospecific females (equivalent to a single teleiochrysalis female), whereas males from both species show strong mate preference for conspecific females (Collins et al. 1993). However, when the concentration was increased (equivalent to 0.4 teleiochrysalis female) or the extract duration was prolonged (from 24 to 48 h), *T. urticae* males were arrested by extract from conspecific females more than by that from *T. evansi*. This result suggests that if the concentration is appropriate, *T. urticae* males are able to discriminate conspecific females from *T. evansi* females by using the difference in pheromones. Therefore, mate preference for conspecific females in *T. urticae* males is supported by their ability to recognize conspecific females using pheromone cues.

On the other hand, the previous study found that *T. evansi* males showed strong mate preference for heterospecific females in choice conditions (Sato et al. 2014a, 2016b). In this study using female pheromone extracts, we found that *T. evansi* males did not respond to extract from either species when the concentration was low (equivalent to 0.2 teleiochrysalis female in 24 h-extract). When the concentration was increased (equivalent to 0.4 teleiochrysalis female) or the extract duration was prolonged (from 24 to 48 h), *T. evansi* males were arrested by the extracts. However, the duration of arrestment was not significantly different between those from conspecifics and heterospecifics. It is unknown if *T. evansi* males start to be arrested by extracts from heterospecifics when the concentration was increased even more. However, the results clearly show that *T. evansi* males are less sensitive to pheromone extracts compared to *T. urticae* males, that is, they are less able to discriminate between con– and heterospecific females by using female pheromones. As *T. urticae* is subject to reproductive interference by *T. evansi* (Sato et al. 2014a), the results meet with the idea that RI is often caused by incorrect mating approaches due to lack of recognition ability in males (Gröning and Hochkirch 2008; Burdfield-Steel and Shuker 2011; Shuker and Burdfield‐Steel 2017). However, this does not explain male mate preference of heterospecific females over conspecifics in *T. evansi* found in previous studies (Sato et al. 2014a, 2016b). In mantids, replacement of indigenous species, *Orthodera novaezealandiae* with exotic species, *Miomantis caffra* was found, and RI is thought to be the mechanism of replacement, because indigenous mantid males are attracted by volatile female pheromone of exotic mantids much more than conspecifics (Fea et al. 2013). In this study, we dealt with contact chemicals, but males may use other types of chemicals such as volatile pheromones, trails on leaves, chemicals on web, and chemicals in faeces. Besides, males may use non-chemical cues as well. In other arthropods, males often use visual cues besides chemical cues to find appropriate females as in the rock shrimp, *Rhynchocinetes typus* and the vine bud moth, *Theresimima ampellophaga* (Díaz and Thiel 2004; Toshova et al. 2013). As eyes of spider mites are primitive and not for forming images but perceive certain wavelengths of light (McEnroe and Dronka 1969; Mills 1974), spider mites are often thought to rely on chemical and tactile cues much more than visual cues to survive (e.g., Sato et al. 2003). However, there is a report that more males approached yellow stimuli sources than non-yellow sources in *T. urticae* (Royalty et al. 1993a). The body colour is different between *T. evansi* and *T. urticae* females: white and green for *T. urticae* and red for *T. evansi* (see Fig. 1 in Sato et al. 2014a). Before concluding that RI occurs because of the inability of *T. evansi* males in female species recognition, it is necessary to investigate other types of chemicals and also non-chemical cues for searching and accepting females in males.

Several studies reported arrestment of *T. urticae* males by female pheromone extracts. The effective range of extract concentration varies among studies. For example, in Cone et al. (1971), extracts equivalent to 0.01 to 0.1 teleiochrysalis females arrested males well. On the other hand, in Royalty et al. (1992), maximum male arrestment was found in the extracts equivalent to a single teleiochrysalis female. In our study, we used extracts which were equivalent to 0.2 and 0.4 teleiochrysalis females, and the extract equivalent to 0.4 teleiochrysalis females was sufficient for *T. urticae* males to discriminate from heterospecific pheromone. As the experimental procedures were slightly different among these studies, the results should not be compared directly. However, as discussed in Royalty et al. (1992), host plant difference in mite culture possibly affect the difference in attractiveness of extracts, as Cone et al. (1971), Royalty et al. (1992) and this study used hops, bean leaves and tomato leaves as host plants, respectively. If host plant difference affects the attractiveness of female pheromone, the effect should be taken into consideration when we think about competitive relationships between *T. urticae* and *T. evansi* in fields, because *T. urticae* infests not only tomato but also various other plants. It would be worthwhile to investigate the effect of host plant and effective concentration.

### Pheromones and female web sharing

Previous study found that *T. evansi* females tend to avoid sharing webs with *T. urticae* females, whereas *T. urticae* females show a preference for sharing webs with *T. evansi* females (Sato et al. 2016a). The propensity of interspecific web sharing in females may affect the probability that females meet interspecific males, and hence the likelihood of RI. Therefore, in this study, we also investigated the effects of female pheromone extracts on females. Females of both species were arrested by extracts from conspecific females when the concentration was low, but the effect disappeared when the concentration was increased. Females of both species were arrested by 48 h extracts regardless of extracted mite species. The results suggest that the females are able to perceive the female pheromones and to discriminate conspecifics from heterospecifics if the concentration is appropriate. Considering that the pheromones can be extracted from protonymph, deutochrysalis, deutonymph, teleiochrysalis females, adult females and slightly from adult males in *T. urticae* (Royalty et al. 1992; Margolies and Collins 1994), females may use the pheromones for understanding their surroundings. Especially, *T. evansi* females tend to share webs with conspecifics and show gregariousness much more than *T. urticae* females. They possibly use aggregation pheromones. On the other hands, if *T. urticae* females use the pheromones for web sharing, they should have been arrested by the extract from *T. evansi* females more than from conspecifics, because *T. urticae* females show a preference for webs of *T. evansi* females over that of conspecifics (Sato et al. 2016a). It is reported that *T. urticae* uses chemicals from its black faeces on silken webs for aggregation, although chemicals from eggs on silken webs do not have such effects (Clotuche et al. 2014). We do not know if the extracts used in this study contain such chemicals from black faeces or not, however, it would be worthwhile to investigate if they use chemicals from black faeces for their decision of interspecific web sharing.

### Variation in duration of arrestment

Both in males and females, variation within treatment in duration of arrestment was large, even in the control treatment. Large variance in data might be inevitable in ethological data. However, in males, the difference in male mating tactics among individuals possibly affected the variance. So far, three male mating phenotypes have been described in *T. urticae*: territorial (= fighting), sneaking and opportunistic tactics (Sato et al. 2013). Territorial and sneaking males spend much time guarding or mounting the teleiochrysalis females. Opportunistic males, however, wander around in search of females that are in the teleiochrysalis stage but very close to or at emergence. Territorial and sneaking males are likely arrested by sex pheromones much longer than opportunistic males. Indeed, some previous studies on female pheromones in *T. urticae* used only males who previously guarded females, because males who did not previously show guarding behaviour showed no arrestment or shorter arrestment by female pheromone extracts (Margolies and Collins 1994). In this study, we did not use males who had previously guarded females, but used virgin males who had emerged into adulthood within 48 h and had no experience of the presence of other mites to remove any possible effect of previous experience. Sneaking tactic is relatively rare but more found in young males (Sato et al. 2014b). Therefore, tested males possibly display any kind of male mating tactics. This can be the reason why the variance in data was large and also why the results were slightly different from previous studies (e.g., the mean arrestment duration) (Royalty et al. 1992; Margolies and Collins 1994), although we should also consider the differences in mite strain, environment and other factors.

In females, the difference in trial start time possibly also contributed to the large variance in the data. We did not see a significant effect of trial start time on the duration of arrestment in males. However, in females, as the start time was getting late, the duration of arrestment tended to be long in *T. evansi* and short in *T. urticae*. We carried out each treatment together with the control treatment, so the conclusion of our study is not affected. However, the results clearly show the importance of time in researches dealing with behaviour.

## Conclusion

We investigated the role of female pheromones on male mate preference and female web sharing to understand the proximate mechanism of RI between Spanish *T. evansi* and *T. urticae* found in previous study (Sato et al. 2014a, 2016b). The difference in mate preference between *T. evansi* and *T. urticae* males may be a key factor in their competitive interaction because it causes unidirectional RI. Results suggest that facilitated heterospecific mating in *T. evansi* males is caused by less sensibility to sex pheromones rather than by higher attractiveness in *T. urticae* female pheromones. On the other hand, mate preference for conspecific females in *T. urticae* males was supported by their high sensitivity to pheromones and ability to discriminate between pheromones of con- and hetero-specifics. Therefore, difference in sensitivity to sex pheromones in males may cause unidirectional RI between them. However, lower sensitivity to sex pheromones does not explain the male mate preference for heterospecifics to conspecifics in *T. evansi*. It is necessary to investigate the involvement of other chemicals and other types of cues. In females, mate preference may be less important for RI, because females rarely succeed in rejecting undesirable male mating approach. However, propensity of web sharing with different species and gregariousness in females may affect the likelihood of RI, because it affects the probability that females meet heterospecific males. Therefore, we investigated if *T. evansi* and *T. urticae* females were arrested by pheromone extracts from con- and heterospecific females, and found that both species females perceived female pheromone and were arrested by conspecific pheromones. The result is inconsistent with previous finding that *T. urticae* females prefer to share web with *T. evansi* (Sato et al. 2016a). Therefore, we concluded that involvement of other chemicals and other types of cues should also be investigated in the female relationships.
